# Editorial: Palliative care for people living with heart and lung disease

**DOI:** 10.3389/fcvm.2022.1127688

**Published:** 2023-01-10

**Authors:** Piotr Z. Sobanski, Małgorzata Krajnik, Sarah J. Goodlin

**Affiliations:** ^1^Palliative Care Unit, Department of Internal Medicine, Schwyz Hospital, Schwyz, Switzerland; ^2^Department of Palliative Care, Collegium Medicum in Bydgoszcz, Nicolaus Copernicus University in Torun, Bydgoszcz, Poland; ^3^Oregon Health and Science University, Portland, OR, United States; ^4^Patient-centered Education, Portland, OR, United States

**Keywords:** palliative care, heart failure, chronic obstructive pulmonary disease, assist devices, end of life

“Suffering is only intolerable when nobody cares” (1).

Palliative care (PC) takes the publication “The Physical and Mental Distress of the Dying” by Hinton in June 1968 as its symbolic starting point (2). Assessing the distress in those who were thought to die within 6 months, he sowed the seed of the idea of providing care focused on limiting the suffering of those approaching death. Hinton stated, based on interviews with 102 patients approaching death, 14 of whom had heart or renal failure, that the physical distress caused by symptoms in people affected by organ failures was more prevalent (especially in respect to dyspnea, nausea/vomiting) and remained more frequently unaddressed (in the case of breathlessness in 82%), than in people dying of cancer. This paper became one of the founding texts for the development of the hospice movement in the UK. As charities financing hospices were focused on supporting care for people dying of cancer, in its first decades PC was almost exclusively limited to this group of patients.

Unfortunately, over 50 years later, the situation has not yet changed substantially. The provision of PC for people living with non-oncological diseases, despite improving in recent years, remains marginal (3). This is despite the fact that, by signing the World Health Organization resolution on Universal Health Coverage in 2019, all countries have an obligation to provide adequate care including PC to all individuals who need it, across their life course, with access to PC recognized as a fundamental human right.^1^,^2^ Cardiovascular disease, cancer, and chronic respiratory disease are considered to be the three most common chronic health conditions causing a high risk of suffering and which may require PC (see text footnote 2).

One of the most relevant steps to provide optimal care (in any discipline, and any health-related problem), is timely recognition of the need of an appropriate intervention. Should it not be the same with respect to suffering? The surprise question (“Would you be surprised if a patient were to die within 1 year”) has been suggested as a trigger for considering the provision of PC to broader groups of patients (Blum et al.) (4). Adding a second surprise question (“Would I be surprised if this patient is still alive after 12 months”) has been proposed in countries with a progressive aging society, such as in the Netherlands, as being more appropriate for the recognition of the probability of a need to start PC based treatment (5). However, as it would not surprise their health care professional if many old and very old people would die, equally it would not surprise them if those in good health were still alive after 12 months. This “surprise” based approach has improved the recognition of the need to begin PC for a higher number of otherwise overlooked people, but it only focuses attention on those at risk of dying. Improving or at least maintaining the best possible quality of life (QoL) must always be the aim in modern health care. A focus on improving QoL should not be delayed until suffering has become unbearable, curative treatment strategies have been exhausted, or the risk of dying has been recognized.

Data from clinical studies and the guidelines of scientific societies recommend the early integration of PC during the course of a disease, concurrent with active treatment in oncology, but this approach is still very much the exception in non-oncological disease (6–10). An Australian group has stated that the need for PC does not correlate with prognosis (11), and should be assessed early in the course of any disease that carries a risk of suffering, using validated tools. This approach is concordant with the different definitions of PC published by globally recognized organizations (e.g., World Health Organization, WHO)^3^ stressing that it should be provided to people living with various diseases which cause health-related suffering (*none* of the current definitions mentions the risk of dying or short life expectancy as criteria warranting the provision of PC). The European Society of Cardiology, in its current guidelines for the diagnosis and treatment of acute and chronic heart failure (HF), states that many patients with HF would derive benefits from the early integration of a palliative and supportive approach (12). The systematic review of accessible needs assessment tools presented in the paper by Waller et al. in this issue gives strong hints as to how to implement needs based provision of PC. Those instruments should be used regularly in all units where care is provided for people living with conditions causing a high-risk of suffering, as discussed in papers by Gonzalez-Jaramillo et al. and Hentsch et al., also in this issue (9).

Palliative Care aims to address all of the dimensions in which needs can emerge: physical (caused by symptoms), psychosocial (caused by emotional distress and social isolation) and spiritual (such as coping with existential dilemmas) (13). It is appropriate with any disease and at any age, thus this issue of Frontiers in Cardiovascular Medicine presents papers on PC with different cardiovascular and respiratory diseases causing significant health-related suffering, (like HF; chronic obstructive lung disease, COPD; interstitial lung fibrosis and congenital heart disease) (Hentsch et al.; Bergsträsser et al.; Janowiak et al.). Some of those with common risk factors can coincide (HF and COPD) causing even more prominent suffering than those diseases alone, as presented in the paper by Kowalczys et al. PC is appropriate for any age group, therefore the paper by Bergsträsser et al. discussing care for children living with advanced HF has also been included in this Research Topic. Other papers discuss breathlessness (presenting a case series of people experiencing the side effects of opioids used for breathlessness alleviation) (Sobanski and Currow), non-pharmacological interventions (Pyszora and Lewko), and spiritual care (Kotlińska-Lemieszek et al.) in people living with COPD.

Spirituality is of great significance for many people, especially when coping with a serious disease and at the end of life. Unfortunately, it is usually overlooked in modern medicine but can be an important resource for many people, including those living with HF (14, 15) or COPD (Kotlińska-Lemieszek et al.). To understand that spirituality is of fundamental significance to most people, it is important to be aware that it is a broader than classically perceived religiosity. The European Association for Palliative Care defines spirituality as a dynamic dimension of human life that relates to the way persons (individuals and in a community) experience, express and/or seek meaning, purpose and transcendence, and the way they connect to the moment, to self, to others, to nature, to the significant and/or the sacred (16). A training program for medical students on spiritual care in medicine implemented in a Polish medical university has been described by Fopka-Kowalczyk et al.

An integral part of PC is to give support in decision making to all involved—patients, their relatives and health care professionals. It should be based on in depth communication, not only focused on informing about the potential benefits to be had from therapeutic options, but also the risk of treatment failure, a burden that needs to be calculated in consent for invasive interventions, the probable progression of the disease, and finally the risk of dying. All of these difficult aspects of clinical communication are usually neglected in conversations between ill people and their physicians (17, 18). End-of-life issues are often omitted, even if life is obviously approaching its end. Open, sensitive communication on imminent death with patients (and their families) can lead to collaborative adjustment of the goals of ongoing therapy. Martínez-Sellés and Grodzicki discuss the matter in this issue of Frontiers, specifically the quality of life driven modification of medical therapy and the activity of implantable devices in patients with advanced HF according to agreed, adjusted goals of care. Therapeutic interventions based on (functional) replacement of an exhausted heart give hope for improving life, but nevertheless come with a high risk of burden and complications. That is why in-depth communication with potential candidates for such treatment is mandatory. This is discussed in the paper by Tenge et al. in a group of patients receiving left ventricular assist devices (LVADs). Ethical challenges and moral dilemmas with respect to the discontinuation of circulatory support are discussed in a paper by Mueller.

Despite undergoing a tremendous evolution in recent years, PC is usually still perceived as a discipline dedicated to those who are dying and are in the last hours of life, when nothing else can be proposed. While much can still be done to improve quality of life during the dying phase, we advocate that disease modifying management incorporate care to assure the best possible quality of life by alleviating symptoms, ensuring compassionate communication, and support for spiritual and existential issues. We hope that this Research Topic of Frontiers in Cardiovascular Medicine will invite you to reflect on how together we can improve care for people with serious heart and lung diseases.

## Author contributions

PZS drafted the manuscript. MK and SJG provided edits. All authors contributed to the article and approved the submitted version.

## Footnotes

^1^Available online at: https://www.who.int/health_financing/universal_coverage_definition/en/ (accessed November 2022).

^2^Available online at: https://www.who.int/news-room/fact-sheets/detail/palliative-care (accessed November 2022).

^3^Available online at: https://www.who.int/health-topics/palliative-care.
